# Isotretinoin-associated Sweet’s syndrome: a case report

**DOI:** 10.1186/s40199-014-0069-2

**Published:** 2014-10-17

**Authors:** Jamileh Moghimi, Daryiush Pahlevan, Maryam Azizzadeh, Hamid Hamidi, Mohsen Pourazizi

**Affiliations:** Department of Internal Medicine, School of Medicine, Semnan University of Medical Sciences, Semnan, Iran; Research Center for Social Determinants of Health, Semnan University of Medical Sciences, Semnan, Iran; Department of Dermatology, School of Medicine, Arak University of Medical Sciences, Arak, Iran; Students’ Research Committee, Semnan University of Medical Sciences, Semnan, Iran

**Keywords:** Sweet syndrome, Isotretinoin, Neutrophilic dermatosis, Drug reaction

## Abstract

**Objective:**

Sweet’s syndrome (SS) is characterized by various clinical symptoms, physical features, and pathological findings. Although cases of SS are very rare, there has been an increase in the incidence of drug-induced SS. Till date, there have been only few reported cases of isotretinoin-induced SS.

**Case summary:**

In this report, we describe the case of a 19-year-old girl who developed SS after systemic treatment with oral isotretinoin for nodulocystic acne.

**Conclusions:**

The findings of this report emphasize the importance of evaluating isotretinoin as a possible, though uncommon, cause of SS and replacing it with another treatment if its involvement is suspected.

## Background

Sweet’s syndrome (SS) is characterized by painful erythematous skin lesions due to neutrophil infiltration in the upper dermis, fever, leukocytosis with a predominance of neutrophils, and increased erythrocyte sedimentation rate (ESR) [1,2]. There are three clinical forms of SS: classical (idiopathic), malignancy associated, and drug induced [3]. Classical SS usually develops in women aged between 30 and 40 years and is often preceded by upper respiratory or gastrointestinal tract infection, inflammatory bowel disease, and pregnancy. Malignancy-associated SS can occur as a paraneoplastic syndrome. Drug-induced SS most commonly occurs in patients who have been treated with granulocyte colony-stimulating factor [3]. Although this form of SS is very rare, there has been an increase in the incidence of drug-induced SS [4]. Here we describe the case of a 19-year-old girl who developed drug-induced SS after systemic treatment with oral isotretinoin (13-cis-retinoic acid) for nodulocystic acne and emphasize the importance of evaluating isotretinoin as a possible, though uncommon, cause of SS.

## Case presentation

**Figure 1:**
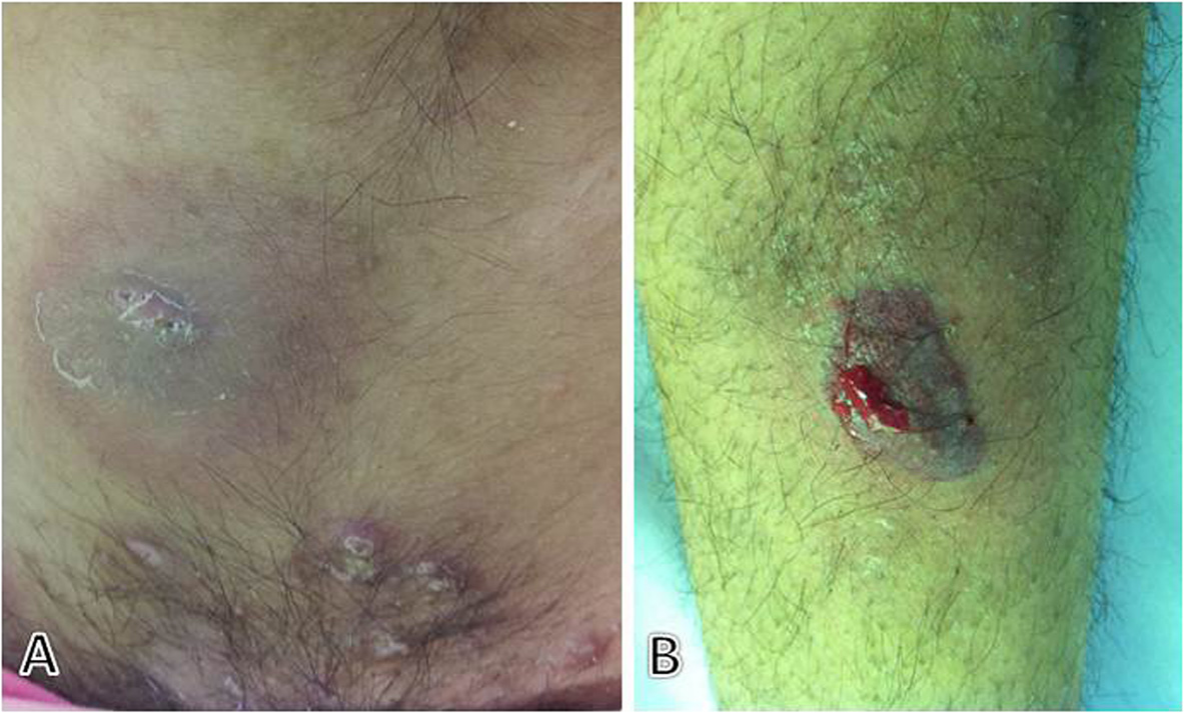
**Isotretinoin-associated Sweet’s Syndrome.** Erythematous, edematous, painful plaques on suprapubic area **(A)** and right lower extremity **(B)**.

**Figure 2:**
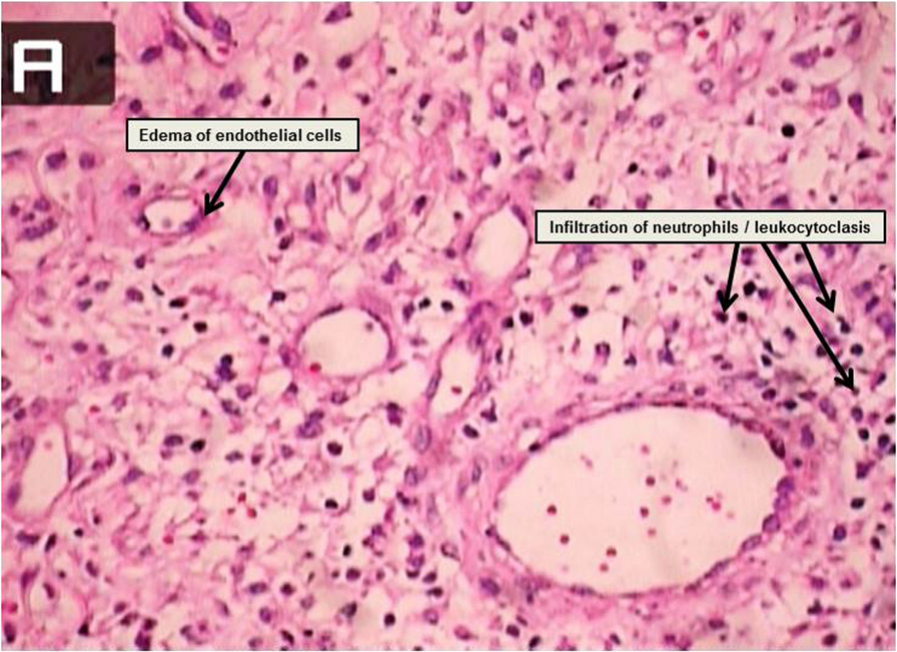
**Isotretinoin-associated Sweet’s Syndrome.** Edema of dermis, infiltration of neutrophils and leukocytoclasis, edema of endothelial cells and no vasculitis.

A 19-year-old girl presented to the rheumatology clinic because of malaise, polyarthralgia, and weight loss (5 kg), which she experienced during six months before presentation to the clinic. She did not have any fever during presentation to the clinic. During two weeks before presentation to the clinic, she developed very painful and tender skin lesions on her legs and in the suprapubic area. Her medical history was insignificant. She had no history of inflammatory bowel disease or rheumatological disease. She did not have symptoms indicative of contact dermatitis or any history of drug intake, except for the use of isotretinoin for severe facial acne vulgaris, which was initiated one year ago at a dose of 20 mg twice daily and was tapered gradually to a dose of 20 mg weekly. A similar lesion appeared 1 month after the initiation of isotretinoin therapy. These lesions resolved without intervention. However, the lesions at the time of presentation to the clinic were more severe and painful, and the patient was admitted to the hospital for clinical evaluation. Physical examination showed that she was unnaturally thin and that she looked ill. Her vital signs were not remarkable, and she did not have fever, lymphadenopathy, hepatosplenomegaly, and other symptoms. Dermatological examination showed tender erythematous plaques with pustules and crust on the ventromedial aspects of her mid-thighs, suprapubic region, and right calf [Figure 1]. Initial laboratory examination determined leukocytosis (23100 cells/mm^3^) and neutrophilia (65%). Group A streptococcal throat culture and anti-streptolysin O titers were negative. Her ESR was >100. Antineutrophil cytoplasmic antibody and antinuclear antibody titers and blood, wound, and urine cultures were negative. Tuberculosis skin test (PPD skin test) provided insignificant results. Kidney and liver function tests, urine analysis, echocardiography, chest radiography, and total abdominopelvic sonography yielded normal results. Bone marrow aspiration and biopsy yielded normal results. Therefore, hematological and solid organ malignancies were ruled out. Skin punch biopsy from a new, well-developed lesion showed spongiotic epidermis with mild dermal edema. Massive aggregates of neutrophils were observed in the upper and mid-dermis, but there was no obvious sign of vasculitis [Figure 2]. Thus, the patient was diagnosed as having drug-induced SS due to isotretinoin based on the above observations and criteria for SS. Isotretinoin therapy was stopped, and prednisolone therapy was initiated at a dose of 60 mg/day (1 mg/kg). Dose tapering was initiated after 2 weeks; after 6 weeks, the dose was tapered to 5 mg/day. Complete resolution of the lesions was observed within 4 weeks. The patient was advised to avoid isotretinoin and asked to return for follow-ups.

### Ethical approval

This is a report of rare case, however the report was approved by the Ethics Committee of Semnan University of Medical Sciences, Semnan, Iran.

## Conclusions

Etiologically, SS may be associated with drugs, infections, and paraneoplastic or inflammatory conditions such as inflammatory bowel diseases and rheumatological diseases. When no triggering factor is detected, the disease is categorized as classic or idiopathic [4].

Only few cases of SS have been reported to be caused by drug reactions. Drug-induced SS is rare and probably represents <5% of all cases [5]. The clinical presentation and histology of drug-induced SS are similar to those of idiopathic SS [6]. Neutrophilia is often absent in drug-induced SS probably because many of these cases are caused by the use of hematopoietic growth factors that are used for reversing chemotherapy-induced neutropenia. Lesions in drug-induced SS usually develop approximately one week after the initiation of the drug [7]. After withdrawing the causative drug, fever abates in 1–3 days and lesions disappear within 3–30 days [5]. Systemic corticosteroids may be required in severe cases. Drugs associated with drug-induced SS include all-trans-retinoic acid and G-CSF or GM-CSF [5]. Some cases may be attributed to trimethoprim/sulfamethoxazole, norfloxacin, furosemide, celecoxib, tetracyclines, and antiepileptic drugs [4,7]. Other implicated drugs are summarized in Table 1 [4].

**Table 1:** **Drugs reported as inducing Sweet’s syndrome**

**Granulocytes growth factors**	G-CSF (granulocyte-colony stimulating factor); GM-CSF (granulocyte monocyte colony stimulating factor); All trans-retinoic acid
**Vaccination**	Calmette-Guérin; Influenza; Streptococcus pneumonia; Small-pox
**Antibiotics**	Doxycycin; Cyclines (minocycline, tetracycline and doxycycline); Quinolones: norfloxacin, ofloxacin; Nitrofurantoin; Streptogramin (quinupristin/dalfopristin); Trimethoprim-sulphamethoxazole
**Antivirals**	Abacavir; Acyclovir
**Anti-tumoural biotherapies**	Proteasomes inhibitors (bortezomib); Tyrosine kinases inhibitors (imatinib)
**Non-steroidal anti-inflammatory drugs**	Diclofenac; Celecoxib; Rofecoxib
**Psychotropes**	Amoxapine; Clozapine; Diazepam; Lormetazepam
**Miscellaneous**	Azathioprine; Carbamazepine; Furosemide; Hydralazine; Isotretinoin; Lenalidomide; Oral contraceptive; Levonorgestrel/ethynil oestradiol; gestodene/ethynil oestradiol; Propylthiouracil

Drug-induced SS is uncommon. Because of the lack of useful and appropriate criteria for its diagnosis, Walker and Cohen proposed five specific diagnostic criteria in 1996 [7]. Thompson and Montarella performed a systematic review of literature on drug-induced SS and evaluated the degree of causal relationships in different case reports [4]. In our case, we used the modified Naranjo criteria to diagnose drug-induced SS [4]. The modified scale includes 10 criteria: presence of previous conclusive reports; temporary onset related to drug administration; temporary resolution of lesions after drug withdrawal or treatment with a specific antagonist; temporary recurrence of lesions due to drug readministration; presence of alternative causes (other than drugs) that could cause the reaction; appearance of lesions after placebo administration; drug detection in the blood (or other fluids) at concentrations known to be toxic; role of drug dosage in the worsening or improvement of the reaction; history of exposure to the same or similar drugs, followed by a similar reaction; and confirmation of an adverse event by any objective evidence [4]. The case reported here had a causality score of 4 based on the above criteria (Possible). This score was not higher because no rechallenge was performed due to the severity of the symptoms. There were no signs of any inflammatory disease, neoplasia, or pregnancy. To the best of our knowledge, this is the third case of SS caused by isotretinoin and the second case of SS caused by isotretinoin therapy for acne vulgaris.

The first case was reported by Gyorfy et al in 2003*.* They reported the cases of two children who developed SS due to isotretinoin administration. However, the children were administered this drug to prevent neuroblastoma recurrence and dysplastic colon re-emergence after bone marrow transplantation and not for acne treatment [8].

The other case of isotretinoin-induced SS was reported by Ammar et al. in 2007 in a 19-year-old man who received isotretinoin for severe acne vulgaris that was resistant to standard topical acne treatment. After one week of the treatment, the patient developed SS. His condition was improved by continuing isotretinoin and by initiating corticosteroids. However, he was diagnosed as having ulcerative colitis two years later [9].

In drug-induced SS, a pre-existing underlying condition associated with SS should be excluded. Clinical criteria can help in distinguishing drug-induced SS from underlying conditions. Based on the observation made in this case, we emphasize the importance of evaluating isotretinoin as a possible, though uncommon, cause of SS and replacing it with other treatments if its involvement is suspected.

## Consent

Written informed consent was obtained from the patient for the publication of this report.

## Abbreviations

SS: Sweet’s syndrome
ESR: Erythrocyte sedimentation rate
